# Comprehensive analysis of the T-cell receptor beta chain gene in rhesus monkey by high throughput sequencing

**DOI:** 10.1038/srep10092

**Published:** 2015-05-11

**Authors:** Zhoufang Li, Guangjie Liu, Yin Tong, Meng Zhang, Ying Xu, Li Qin, Zhanhui Wang, Xiaoping Chen, Jiankui He

**Affiliations:** 1 Department of Biology, South University of Science and Technology of China, Shenzhen 518055, China; 2 State Key Laboratory of Respiratory Disease, Center for Infection and Immunity, Guangzhou Institutes of Biomedicine and Health, Chinese Academy of Sciences, Guangzhou 510530, China; 3 Department of Infectious Diseases and Hepatology Unit, Nanfang Hospital, Southern Medical University, Guangzhou 510515, China

## Abstract

Profiling immune repertoires by high throughput sequencing enhances our understanding of immune system complexity and immune-related diseases in humans. Previously, cloning and Sanger sequencing identified limited numbers of T cell receptor (TCR) nucleotide sequences in rhesus monkeys, thus their full immune repertoire is unknown. We applied multiplex PCR and Illumina high throughput sequencing to study the TCRβ of rhesus monkeys. We identified 1.26 million TCRβ sequences corresponding to 643,570 unique TCRβ sequences and 270,557 unique complementarity-determining region 3 (CDR3) gene sequences. Precise measurements of CDR3 length distribution, CDR3 amino acid distribution, length distribution of N nucleotide of junctional region, and TCRV and TCRJ gene usage preferences were performed. A comprehensive profile of rhesus monkey immune repertoire might aid human infectious disease studies using rhesus monkeys.

T cell receptors (TCR) are protein complexes on the cell surface of T lymphocytes that play key roles in adaptive immune responses. They are heterodimeric molecules, and greater than 95% of TCRs in the circulation belongs to the αβ type. In humans, there are more than 2 × 10^7^ unique TCRα and TCRβ pairs in the peripheral blood of an individual^1^,^2^. The complexity of the immune repertoire is generated by genomic rearrangement of Variable (V), Diversity (D), Joining (J) gene segments during the maturation of lymphocytes. The joining region of the VDJ gene segments is the major antigen recognition site, also called complementarity-determining region 3 (CDR3), which represents the most diverse and complex region of the variable region.

The rhesus monkey (*Macaca mulatta*) is employed in animal model systems for a number of important human diseases in which T lymphocytes play a central role in pathogenesis. Therefore, characterization of the TCR sequences of this nonhuman primate is very important. Early in 1992, Gene Levinson *et al.* sequenced 23 rearranged TCRβ cDNA clones derived from peripheral blood of a rhesus monkey^3^. Later, Emma E. M. Jaeger further expanded the TCRβ pool in this species^4^. Sequencing alignment of rhesus monkey, chimpanzee and human showed that the diversity, structure and evolution of TCRβ gene repertoire in primates are closely linked with each other^4^,^5^,^6^,^7^,^8^. These works and others totally identified more than 23 V beta genes in rhesus monkey and established a public sequence library of TCRβ sequences, which is critically important for further studies^9^. However, due to the low throughput nature of traditional cloning technology, the TCR sequences revealed in the past two decades in rhesus monkey are only a tiny fraction of the total TCR repertoire.

In recent years, a powerful new technology based on next generation sequencing has been developed to probe the adaptive immune system. Millions of TCR sequences can be amplified in a single multiplex PCR reaction, prepared and then read in parallel from a single sample. In 2009, Weinstein *et al.*, applied high throughput sequencing to study the zebrafish antibody repertoire and estimated that there is between 1,200 and 3,700 antibody sequence per fish^10^. Using a similar approach, Freeman *et al.*, identified 33,664 distinct TCRβ sequences in a human subject^2^. Wang *et al.*, observed the diverse TCRβ repertoire expressed by a healthy human included 113,290 unique TCRβ CDR3 nucleotide sequences^11^. Previous studies have estimated the total diversity of the TCR repertoire to be approximately 10^6^ based on statistical models^2^,^11^,^12^. Dynamic changes of antigen receptors are also observed, particularly among different age groups^13^,^14^,^15^. Recently, this technology has been developed to investigate various human diseases, monitor disease progress, and study the lymphocyte maturation process^16^,^17^. For example, the high sensitivity of this technology enables a more reliable estimation of minimal residual disease in various leukemias^18^,^19^,^20^ and the evaluation of the effectiveness of immunotherapies^21^,^22^,^23^,^24^. This approach is also used to investigate the immune system in cancers^19^,^25^,^26^, infectious diseases such as dengue^27^ and autoimmune diseases^25^, particularly rheumatoid arthritis^28^. Reddy *et al.*, and DeKosky *et al.*, applied immune repertoire sequencing for monoclonal antibody production and demonstrated it produced monoclonal antibodies much faster and cheaper than conventional methods^29^,^30^.

Despite the extensive application of immune repertoire sequencing technology in humans, the usage of this powerful technique in other model organisms is limited to mice and zebrafish^10^,^31^. Rhesus monkey is an important animal model for the study of human diseases. More than 70 infectious diseases have been investigated in the rhesus monkey model^32^,^33^, most notably acquired immunodeficiency syndrome (AIDS) caused by human immunodeficiency virus (HIV)^34^,^35^,^36^,^37^, tuberculosis (TB) caused by mycobacterium^38^, and malaria caused by human *Plasmodium* species^39^.

Here, we established a platform to characterize the TCRβ repertoire of the rhesus monkey immune system using massive parallel sequencing. By analyzing millions of TCR sequence reads, we found that the TCR repertoire of rhesus monkey is highly diversified and the size of TCR repertoire is close to that in human.

## Results

### Sample preparation and sequencing

**Figure 1:**
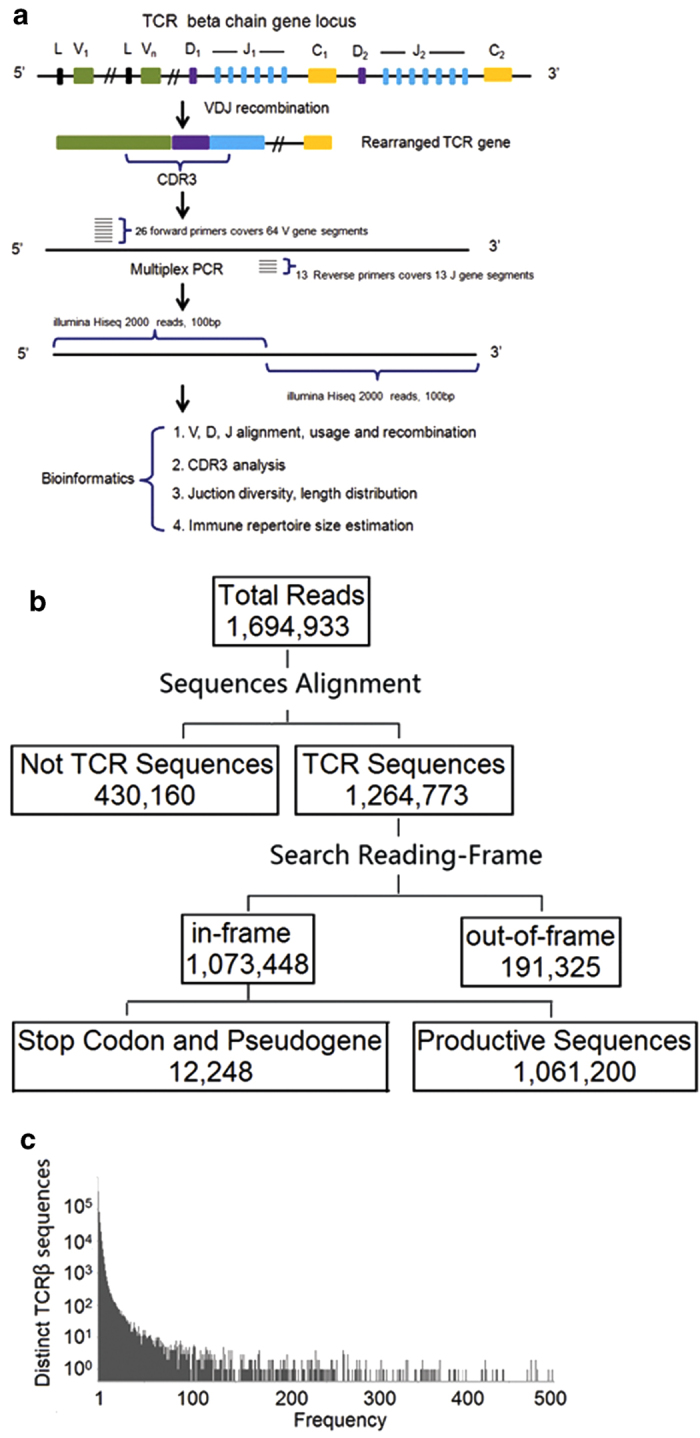
Experimental design and summary of high throughput sequencing data (**a**) Schematic diagram of the TCRβ VDJ recombination, TCR library construction and bioinformatics pipeline in rhesus monkeys. The diversity of TCRβ is generated by VDJ rearrangement and junctional diversity. We designed multiplexed primer sets to amplify sequences including the TCR CDR3 region, which were then sequenced and analyzed. (**b**) Statistical analysis of sequence data. (**c**) Frequency histograms of TCRβ sequences.

We developed an approach to characterize the TCRβ repertoire of rhesus monkeys by analyzing the beta chain, which contains the majority of TCR diversity (Fig. 1a). We isolated peripheral blood mononuclear cells (PBMCs) and extracted genomic DNA from 10 ml blood from two rhesus monkeys. One is for estimating the size of immune repertoire and the other is for validating PCR bias and sequencing errors. Both rhesus monkeys were 10-year-old, female. We designed 26 forward primers in the FR3 region of V gene region and 13 reverse primers (including one degenerate primer) in the J gene region to cover the majority of TCRβ VDJ combinations. The TCRβ library was amplified using genomic DNA as a template by multiplex PCR. The amplicons were approximately 120 base pairs long and were prepared for pair-end sequencing. The first monkey is sequenced at very high coverage using Illumina Hiseq 2000 platform to study the total size of TCR repertoire. Sequence reads were analyzed using our in-house developed Immune Repertoire Analysis Pipeline (iRAP, http://www.sustc-genome.org.cn/irap2/), which is available for online use.

### Statistical analysis of TCR sequences

Approximately 1.69 million clean reads were generated for the first monkey. After aligning to the TCRβ germline reference sequences, 1.26 million sequences were identified as TCRβ sequences (Fig. 1b). Among them, 1.07 million sequences were in-frame and 0.19 million reads were out-of-frame in their CDR3. The ratio of in-frame to out-of-frame was 5.6:1.0. Many T cells that contained out-of-frame TCR sequences and premature-stop-codons might have been eliminated by multiple pathways *in vivo*^40^. These T cells, if not inactivated or destroyed by nonsense-mediated decay (NMD) could encode truncated proteins and cause serious diseases^41^. CDR3 is at the center of the antigen-binding site, which has direct contact with the MHC bound peptide. It is also the most diverse region of the TCRβ gene, and can recognize thousands of different antigens. We identified 0.23 million unique CDR3 amino acid sequences in the sample. In humans, the size of unique CDR3 of TCR repertoire was reported more than 30,000 from 165L of blood pooled from 380 males and 170 females using Illumina GAII analyzer in 2009^2^, and around 0.10 million from one human donor using 454 sequencing platform in 2010^11^, as well as around 0.1 million unique CDR3 sequences from CD4 T cells isolated from 5 ml of blood of a healthy human donor in our unpublished data. The size of rhesus monkey CDR3 sequence diversity identified in this study is comparable to that in humans^12^. The relative frequency distribution of TCR sequences showed that around 25.2% of TCR sequences only had a single copy (Fig. 1c). The overall TCRβ CDR3 oligoclonality index (OI) was 0.63, indicating significant non-uniformity of the frequency of distribution of CDR3 sequences^42^.

### V, D, and J gene segments appear with unequal frequency in rhesus monkeys

**Figure 2:**
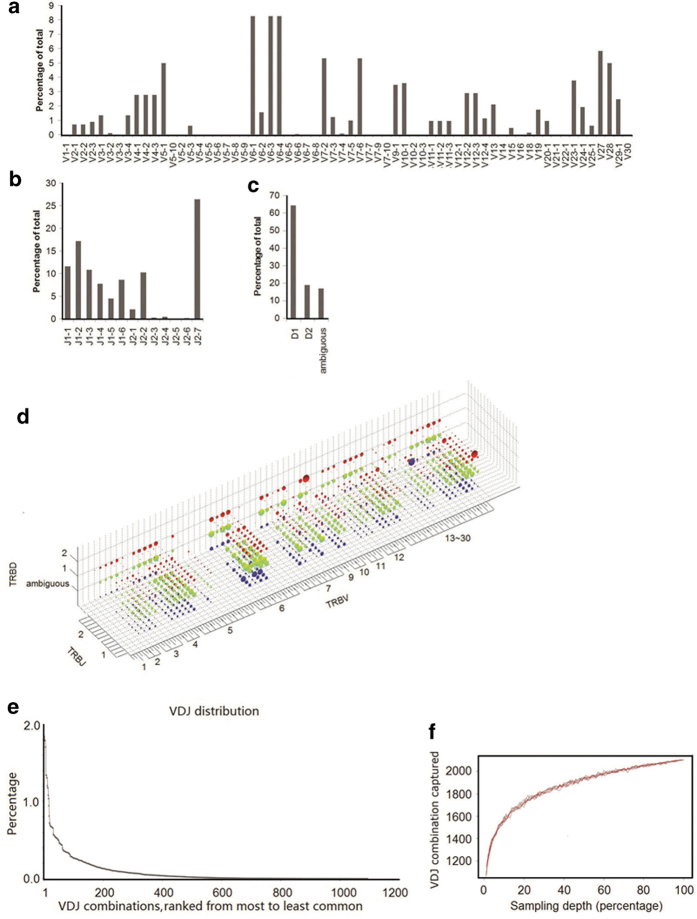
VDJ gene segments usages of TCRβ chain (**a-c**) The TCRβ V, D, J gene segment usage in primary rearrangements. (**d**) The entire VDJ repertoire in a 3D plot. The three axes enumerate all possible V, D, and J values, so each point in the 3D space is a unique VDJ combination. The volume of the sphere at each point corresponds to the number of reads matching that particular VDJ combination. The dot volume is plotted on a linear scale. The red, green and blue color dots correspond to D2, D1 and ambiguous D, respectively. The TCR D gene is the most complex region, and undergoes sustainable deletion and transformation. It is possible many D gene sequences were deleted during VDJ recombination. Therefore, approximately 16.8% of the reads are ambiguous and cannot be assigned to a particular D gene segment. (**e**) Unevenly-distributed VDJ combinations ranked from most to least common. (**f**) Rarefaction studies of VDJ combination. We randomly chose a certain percentage of reads from the total sequence library and calculated the number of VDJ combination captured. We performed the sampling process 10 times for each data point.

The rhesus monkey has 64 V, 2 D and 13 J gene segments in germline references of the international ImMunoGeneTics information system (IMGT) database^43^. Each sequence read was assigned to a particular V, D and J by alignment to the germline references^44^,^45^. We identified 57 out of 64 V types, all 2 D types and all 13 J types in the first monkey. Therefore our multiplex PCR primers cover most of the V and J types and the majority of V, D and J gene segments were detected in this sample. V, D and J gene segments were observed at unequal frequencies, with some segments appearing more frequently than others, resulting in a highly skewed distribution (Fig. 2a–c). The most abundant V gene segments were V6-1, V6-3 and V6-4. The top 8 V gene segments accounted for 50% of all clusters. The uneven usage pattern was also observed for J gene segments, with J2-7 accounting for 27% of all J gene segments. We also observed that D1 had a higher frequency than D2. This phenomenon can be explained by proximal efficiency during recombination^31^,^46^. There are overall 65 V genes types in human, however, similar to our observation, the usage of different V genes are not equal. For example, in year 2009, 49 V genes are identified out of 64 V types in humans and the most abundant V gene families observed in their work including V20-1, V5-1 and V29-1^2^.

### VDJ combinations appear with unequal frequency in rhesus monkeys

There are 1,664 possible VDJ combinations in rhesus monkey (64 V × 2 D × 13 J = 1,664 VDJ). We observed 1,094 VDJ combinations in the first monkey (Fig. 2d, Supplementary Fig. S6). The VDJ combination coverage was 66%. Using subsets of full data sets to perform rarefaction studies, sampling of the VDJ repertoire was directed towards saturation (Fig. 2f). Because V, D and J segments appeared with unequal frequency, we expected unique VDJ combinations to also appear with unequal frequency. Indeed, frequencies ranged from 0.01% to 1.85% for the 1094 different VDJ combinations was observed. The most abundant VDJ combination (V10-1/D2/J2-7) had 12,057 sequence reads, whereas the least abundant VDJ combination had only one sequence read (Fig. 2d). Frequencies of the VDJ combination displayed a long tail distribution (Fig. 2e). The reasons for unequal frequencies of VDJ combinations are not clearly understood, but might due to a combination of proximity effects^46^ and recombination signal sequence compatibilities that influence initial TCR development, plus thymic selection^47^,^48^ and immune challenge^49^,^50^ that modify the representation of selected clones in the repertoire. The possible VDJ combinations are close to that in humans which includes 65 V, 2 D and 13 J as derived from IMGT^43^.

### CDR3 characterization in rhesus monkeys

**Figure 3:**
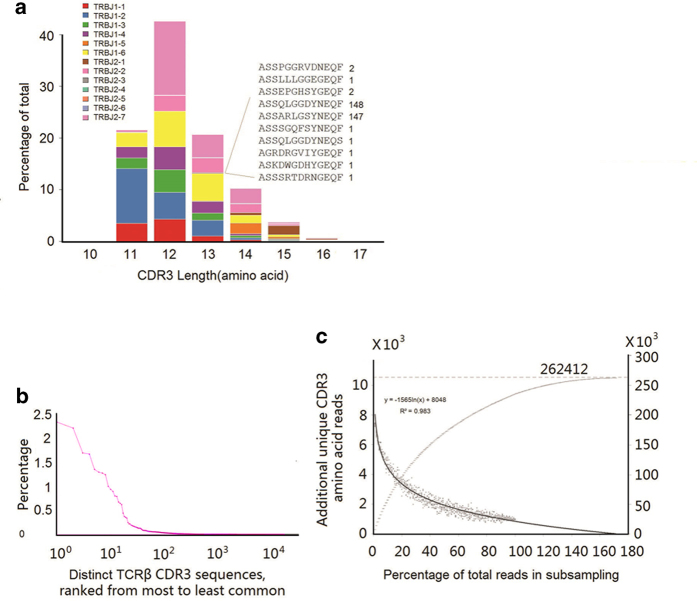
CDR3 characterization of TCRβ (**a**) A representative TCRβ CDR3 sequence spectratype. The sequences within each length bin were color-coded based on their J gene use. The inset shows all CDR3 sequences using J2-1, with a length of 13 amino acids and the number of times that each of these sequences was observed in the data. (**b**) Frequency histograms of TCRβ CDR3 sequences. (**c**) Sampling-resampling techniques were used to estimate the CDR3 repertoire size (described in “data analysis” of the Methods).

One amino acid change in the CDR3 length can lead to conformational remodeling of the receptor. Hence, junction diversity can also be represented by CDR3 length distribution^50^. The CDR3 in rhesus monkey was defined with the same criteria as that in human, which is in between the last cysteine of TRBV and the phenylalanine in the TRBJ segment motif FGXG. The length of CDR3 ranged from 10 to 16 amino acids (30 to 48 nucleotides) and 84% of the CDR3 ranged from 11 to 13 amino acids (Fig. 3a). In humans, the CDR3 length ranges from 7 to 19 amino acids, with the majority between 10 and 14 amino acids^12^. Statistical analysis indicates that the majority of the distinct CDR3 sequences contain less than 10 copies (Fig. 3b).

### Estimate of total TCRβ CDR3 diversity in rhesus monkeys

The diversity of the CDR3 sequence is essential for maintaining normal immune responses. Previous studies suggested the potential size of unique CDR3 in TCRβ of humans is about 340,000^11^. To determine whether our sequencing depth was sufficient to cover the majority of unique CDR3 of the species, we applied sampling-and-resampling techniques to estimate population sizes and diversity. This estimation approach was first developed by Fisher RA *et al.*, and has been widely tested and improved upon by other scientists^1^,^12^,^51^. Briefly, it estimates the diversity of the infinite library using a random, finite sample. In our sample, the actual size of unique amino acid CDR3 sequences observed was 235,573, compared with the potential size of unique CDR3 sequences predicted by sampling-and-resampling that was 262,412 (Fig. 3c). Therefore, according to this estimation, our experiment successfully captured 90% of the total TCRβ CDR3 sequences.

### Junction diversity characteristics in rhesus monkeys

**Figure 4:**
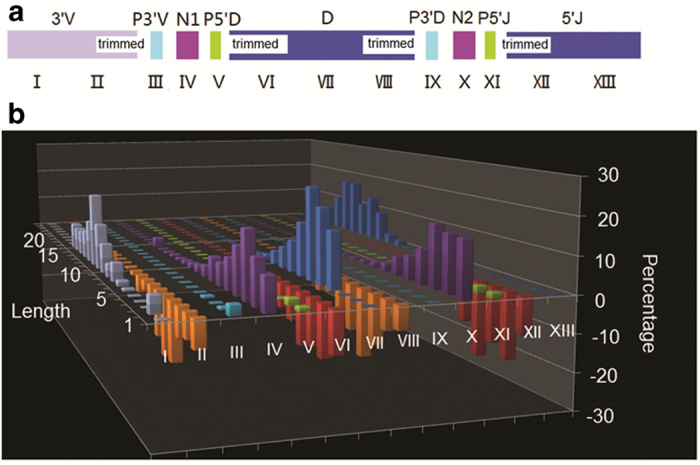
Junction analysis of CDR3 (**a**) Schematic diagram of 13 sections of the junction region. (**b**) The length distribution of insertions and deletions in 13 sections of the junction region. The x-axis is the 13 sections of the junction region and the y-axis is the length of each section added or deleted during the recombination process. The z-axis is the percentage of sequences with a particular length.

Diversity of the T cell repertoire is not only a consequence of different V, D and J rearrangements. Much of the TCRβ diversity is derived from template-independent insertion or deletion of nucleotides at the V-D and D-J junctions within the CDR3 by terminal deoxynucleotidyl transferase^52^. Here we categorized the junction region of rhesus monkey TCRβ into 13 sections (Fig. 4a), as defined by IMGT. We performed statistical analysis of each section for all 270,557 CDR3 nucleotide sequences and plotted their length distribution (Fig. 4b, Supplementary Fig. S4). The mean length of N1 and N2 insertion were 4 base pairs and 3 base pairs, respectively. Although the diversity of TCR genes is primarily generated by VDJ segment rearrangement, our study indicates that CDR3 diversity is mainly dependent upon insertion and deletion in the junctional region.

### PCR bias assessment

**Figure 5:**
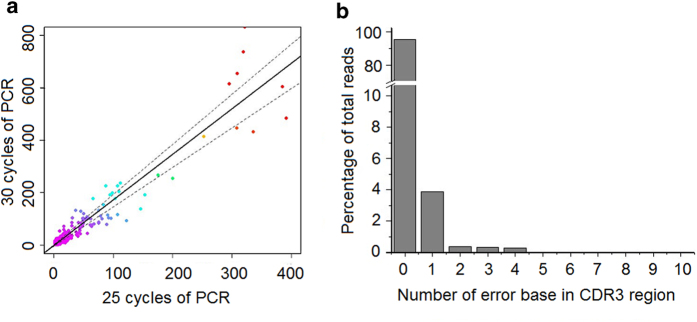
PCR bias and sequencing error rate assessment (**a**) Assessment of PCR bias. The rearranged TCRβ CDR3 sequences present in T-cell genomes were amplified through 25 cycles and 30 cycles of PCR, respectively, each setting with a triplication. Each point on the graph represents a single unique CDR3 sequence, plotted according to the number of times that sequence was observed in the data from 25-cycle and 30-cycle PCR reactions, respectively. The density of sequences at each point in the plot is indicated by different colors, with purple the highest density and red the lowest. The solid line represents a linear regression of the data, plus the dotted lines 1 SD above and below the mean. (**b**) Sequence error estimation. Error rate profile of the data using cloned plasmid library as the references sequence library. Sequence error in each CDR3 sequence was plotted against its percentage in total reads. The error reads accounts for 4.9% of total sequence reads with the error rate in CDR3 is 0.186% per base.

A major objective of this study was to determine the relative abundance of TCR sequences. However, using PCR to amplify the TCRβ CDR3 could potentially introduce a systematic bias in the inferred relative abundance of the sequences, due to differences in the efficiency of PCR amplification of CDR3 using different V and J gene segments. Several error-correcting approaches were previously presented by several studies using different algorithms^10^,^12^,^53^ To estimate the magnitude of any such bias, we followed the methodology introduced by Robins *et al*^12^. We extracted the genomic DNA from the second monkey, prepared the multiplex PCR master mix, and split it equally into 6 tubes, three of which were amplified through 25 PCR cycles, and the other 3 tubes are amplified through 30 PCR cycles. Each PCR products were indexed separately during the sequencing library construction and then sequenced by Illumina pair-end sequencing strategy. Of the 10,345 unique TCRβ CDR3 sequences observed in the 25-cycle PCR experiment, 15.3% were also found in the 30-cycle PCR experiment. We plotted the number of observations of these 19,658 sequence reads in the 25-cycle against the number of 32,647 observations in the 30-cycle, a linear correlation was observed (Fig 5a). For sequences observed a given number of times in the 25-cycle experiment, The variance around the mean of the number of observations at 30 cycles could be due to a combination of PCR bias and sampling variance. Conservatively attributing the mean variation about the line (1.18-fold) entirely to PCR bias, each cycle of PCR amplification potentially introduces a bias of average magnitude 1.033. Thus, the 30 cycles of PCR currently used in our protocol for preparing CDR3 for Illumina sequencing potentially introduces a total bias of average magnitude 1.033^30^ = 2.70 in the inferred relative abundance of distinct CDR3 sequences. The PCR bias in this study is comparable to the study of human immune repertoire by Robins *et al.*^12^.

### Reproducibility

We evaluated the reproducibility of our methods by comparing the results of five experiment replicates from a second monkey. We draw 10 ml blood from the second monkey. The genomic DNA was extracted and split into five tubes. The libraries were constructed following the same protocol and sequenced by Illumina pair-end sequencing strategy. The V gene and J gene usage in 5 replications was highly similar (Supplementary Fig. S1, Supplementary Table. S2). We performed pearson correlation of reads of CDR3 sequences using two representatives replicates S3 and S5. It showed high correlations between the two replicates with correlation coefficient of 0.99 (Supplementary Fig. S2). The results of these two replicates are linearly correlated, indicating that the method is reproducible.

### Sequence error assessment

We then examined the sequences error by using a known set of plasmids derived from the monkey TCRβ sequence. We cloned 26 TCRβ CDR3 sequences, mixed them equally, used them as the templates and performed 20, 25 and 30 cycles of PCR amplification. All sequencing data were pooled together to calculate the error rate. Using the original plasmid sequence as the benchmark, we calculated the error rates. 95.1% of total sequence reads are perfectly matched to the original plasmid sequence with no error, and 4.9% of total sequence reads have one or more sequencing error in their CDR3. We estimated that the error rate in CDR3 is 0.186% per base (Fig. 5b).

## Discussion

Here we established a high-throughput sequencing based platform to study the immune repertoire of rhesus monkeys of the TCRβ gene. We performed comprehensive analysis of VDJ gene usage patterns, VDJ gene segment rearrangements, CDR3 length distribution, CDR3 amino usage patterns, CDR3 size estimation and junction N-nucleotide characterization. We identified 270,557 unique rhesus monkey TCRβ CDR3 gene sequences, which is three orders of magnitude to that in the IMGT and NCBI collections. Overall, the diversity identified in rhesus monkey are comparable to that in human^11^.

The method in this study has been validated, including the PCR amplification bias, reproducibility, and sequencing error. However, the coverage of primers used in this study may need further validation in more samples. We identified 57 out of 64 V types, 7 V types were missing in our dataset. There are three possible explanations. First, this monkey may not express these 7 unobserved V types due to monkey-to-monkey variations. Second, the sequencing is not deep enough to cover the rare V types. Third, these 7 unobserved V types are not amplified by PCR due to the primer problem. We test the primer set in the second monkey, we successfully identified 63 out of 64 V types, which indicated that our primers covers the majority of V gene family (Supplementary Fig. S3). However, a large test in more monkey samples will help to identify the problem. Bias and errors are two problems in immune repertoire studies. Plasmid sequences were used to estimate that 4.9% of CDR3 sequences contained one or more errors. The errors will artificially increase the size of immune repertoire, resulting in overestimation of diversity. We run a computer simulation to estimate to what extent the added sequencing errors will increase the diversity of immune repertoire. By adding 4.9% errors to the original data, the diversity of TCR increased 1.29 times (Supplementary Fig. S7). Several groups have proposed methods to reduce the errors and biases, such as using “random barcode technique” during the experimental design^54^ or by calculating the consensus sequences during the data analysis^55^.

The immune repertoire sequencing has great potential to be widely used to study immune-related diseases in monkey model, which are currently used to investigate over 70 infectious diseases. The immune repertoire sequencing can be applied for a number of applications including tracking the decay of CD4 T cell clones during HIV infection, investigating T cell response during virulent influenza virus infection, studying the mechanisms of T cell responses to mycobacterium infection and facilitating vaccine development for malaria.

## Methods

### Animals

Rhesus monkeys were provided by the Guangzhou Institutes of Biomedicine and Health. The two monkeys used in this study were 10-year-old, female.

### Ethics

Rhesus monkeys were housed at the Non-human Primate Animal Center in the Guangzhou Institutes of Biomedicine and Health (GIBH). Experiments were performed in accordance with the Guide for Care and Use of Laboratory Animals. Animal protocols were approved by the GIBH Institutional Animal Care and Use Committee.

### PBMC isolation

PBMCs were isolated using LymphoPrep™ (Axis-shield, Dundee, Scotland, UK) with slight modification of the manufacturer’s instructions. Briefly, 10 ml of blood was diluted with an equal volume of PBS, loaded carefully onto 5 ml of LymphoPrep (sample to lymphoprep media, 2:1) and centrifuged at 600 × *g* for 20 minutes. The thin PBMC layer at the sample/medium interface was carefully aspirated and transferred to a new tube and washed twice with PBS at 300 × *g* for 10 minutes. The purified PBMC cells were immediately processed for CD4^+^ and CD8^+^ T cell isolation.

### Genomic DNA extraction

Rhesus monkey genomic DNA was extracted using PureLink Genomic DNA Mini Kit (Life Technology, Cat. No: K1820-00) according the manufacturer’s instructions. Briefly, RNAs in PBMCs were digested and 1 volume of PureLink cell lysis buffer with protease K was added to the mixture and incubated for 10 minutes at 55 °C to promote protein digestion. Then, 1 volume of 96% ethanol was added to the mixture to a final volume of 640 μl. The mixture was loaded on to a PureLink spin column. Genomic DNA bound to the column and after several washing steps, the genomic DNA was eluted in elution buffer.

### Sequencing library preparation

To prepare the TCRβ library, a set of forward primers and reverse primers were designed to amplify the CDR3 of the TCRβ gene from the rhesus monkey genomic DNA (Supplementary Table S1). The rearranged TCR fragments was amplified in a 100-μl PCR reaction containing 1× QIAGEN Multiplex PCR master mix (QIAGEN, Valencia, CA, USA), 0.5× of Q-solution, 40 nM of each VF (forward primers designed at V gene region) and JR primer (reverse primers designed at J gene region) (Invitrogen), 4 μg of PBMC gDNA (genomic DNA, ~1.33 × 10^7^ genomic DNA templates). The following thermal cycling conditions were used in a GeneAmp 2700 cycler (Applied Biosystems, USA): one cycle at 95 °C for 15 min, 25 cycles at 94 °C for 30 s, 65 °C for 90 s and 72 °C for 30 s, followed by one cycle at 72 °C for 5 min. The target 120-bp amplicons were purified by Agencourt AMPure XP size selection. One hundred microliter of PCR product were mixed with 120 μl magnetic beads and incubated at room temperature for 5 min; the supernatant was transferred to a new tube and mixed with additional 60 μl of magnetic beads; the magnetic beads were washed twice with 80% ethanol and dried. The amplified DNA fragments were eluted in 40 μl of TE buffer. Around 1 μg of the TCRβ library DNA was sent to BGI-Shenzhen for the Illumina sequencing platform. The sequencing data was analyzed by an in-house immune repertoire analyzing pipeline, including V, D, J assignment, CDR3 length distribution, clustering and other analyses.

### Primers design

The rhesus monkey TCRβ sequences were downloaded from IMGT^43^. Alignment was performed for all 64 V and 13 J genes. A relative conserved region in the frame region 3, upstream of CDR3 was selected as putative forward primer region. A cluster of primers corresponding to the majority of the V gene sequence family was selected. Similarly, reverse primers corresponding to 13 types of the J gene family were designed. The forward and reverse primers were analyzed by Oligo 7.0 and MFEprimer-2.0^56^ for primer dimer and loop structures. Minor changes of the sequences were made for low-quality primers. The final primer sequence is shown in Supplementary table S1.

### Control plasmid library construction

We cloned TCRβ sequences that cover the majority of 64 V gene families. Briefly, genomic DNA of one monkey was extracted and multiplex PCR was performed using the same protocol as described in sequencing library preparation section with the primer set we designed. The PCR product was then cloned using TOPO® TA Cloning Kit for Sequencing (Invitrogen, Carlbad, CA). 26 clones covers 26 V beta gene were picked out and the corresponding plasmids were further expanded and purified. These 26 clones are pooled in equal amount to generate a master mix as the control plasmid library.

### Primer and PCR amplification validation

We validated the primers and the amplification efficiency using both the control plasmid library and the genomic DNA isolated from PBMCs of a healthy rhesus monkey. For the plasmid library, we used the library as the template and amplified them in triplicate by the primer set for 15, 20 and 25 cycles. The PCR products were separated on 2% agarose gel and a band corresponding to 100 to 150 bp was excised and purified using QIAquick Gel Extraction Kit (Qiagen, Valencia, CA). These nine libraries were index separated and pooled together and sequenced by Illumina pair-end sequencing strategy. These nine libraries were index separated, pooled together and sequenced by Illumina sequencing platform. The sequencing data were analyzed using a similar method as proposed by Robins *et al.* to validate the PCR bias^12^. Furthermore, the pooled sequencing data from the control plasmid can be further used to estimate sequencing error rate. Using the original 26 plasmids from Sanger sequencing as the benchmark, the sequencing error were identified and the sequencing error were calculated. Using the same primer set, PCR bias are also checked using the genomic DNA as the template.

### Data analysis

A total of 2,645,432 read pairs were generated by the Illumina paired-end sequencing strategy. The length of each read in one pair is 100bp. All paired-end reads with overlapping bases were merged through FLASH^57^ software (parameter: -x 0.3 -m 10 -M 100) . The reads were obtained from the merged file from FLASH result, and we got 1,694,933 full-length nucleotide reads. The merged sequence reads were aligned to V, D and J gene germline references by IMGT-High V-QUEST^44^ with default parameters. In IMGT-High V-QUEST program, our sequences were firstly aligned to the IMGT V gene reference library, then J genes reference library and D genes references. The V, D and J region are identified. 1,264,773 (74.6%) reads were successfully aligned to V, D and J reference libraries, with alignment identity above 60%. Reads with low alignment identity to germline references were excluded from further analysis (identity of TRBV <60% or identity of TRBJ <60%). The starting and ending position of the junction, CDR3, reading frame, and productivity were identified according to the definition of IMGT. The sequence in junction region is between the conserved amino acid (cysteine 2nd-CYS, C) at position 104 in V region and the conserved amino acid (phenylalanine J-PHE, F) in J region at position 118.

We counted the reads number of each TRBV/D/J genes to calculate the frequency distribution. Some sequences are aligned multiple V genes, due to the diversity of junction. If a sequence is aligned to n V genes, then, each V genes will get 1/n. The distribution graph of TRBV/D/J gene usage was generated on the reads number of each genes by Microcal Origin 7 software. The 3-D plot of V-D-J recombinations was generated on the reads number of each combination type, via Matlab 3Dplot function.

A sampling-resampling technique was performed to predict the size of the immune repertoire. Each time we randomly added one percent of our total TCR reads into our random sample. Then, we calculated the unique CDR3 sequences in our random sample and observed the increasing ratio. In the first subject, the first 1% percent of total CDR3 sequences that we obtained was containing 953 unique CDR3 sequences. The next set of 1% added another 885 unique CDR3 sequences; the third set added another 831, and so on. The unique CDR3 sequences were approaching to saturation. We fitted the pattern with logarithmic decay (R^2^ = 0.97), into a logarithmic curves. This allowed us to calculate the stop-point, at which no additional unique CDR3s would be observed. After that, we built the cumulative curve of number of unique CDR3s, and using the stop-point to calculated the possible size of TCR CDR3 sequences (amino acid).

We used oligoclonality index (OI, see equation (1)) to measure non-uniformity of the frequency of CDR3 sequence distribution, as Weinstein described in 2009. A population with a perfect monoclonal distribution (including only one unique CDR3 sequence), will have an OI of 1. A population with a perfect polyclonal distribution (each unique CDR3 sequence has equal abundance) will have an OI of 0. The OI in (1) is defined based on the Gini coefficient:


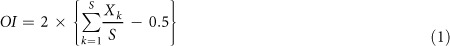


where

***S*** the total number of unique TCRβ CDR3 clones

*S*_*i*_ the abundance of a given TCRβ CDR3 clone


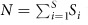
 the read number of TCRβ CDR3 clones


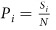
 the relative abundance of TCRβ CDR3 clones


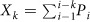
 the cumulative relative abundance of TCRβ CDR3 clones in decreasing order of abundance.

We used the cloned plasmids sequences to calculate the sequencing error. 26 TCRβ genes were cloned into plasmids, and sequenced by Sanger method. The Sanger sequencing is used as references. The sequences reads from multiplex PCR and high throughput sequencing are aligned to the 26 plasmid sequences to validate the sequencing errors. 95.1% of total sequence reads are perfectly matched to the original plasmid sequence with no error, and 4.9% of total sequence reads have one or more sequencing error in their CDR3 region. Among 13,689,229 total sequence bases, 25,534 bases are errors. So the error rate is 0.186% per base.

## Author Contributions

Design the project, J.K.H., X.P.C., Z.F.L.; Perform the experiments: Z.F.L., G.J.L., M.Z., Y.X.; Data Analysis: Y.T., Z.F.L., L.Q. Write and the manuscript: Z.F.L., Y.T., J.K.H., Z.H.W.

## Additional Information

**How to cite this article**: Li, Z. *et al*. Comprehensive analysis of the T-cell receptor beta chain gene in rhesus monkey by high throughput sequencing. *Sci. Rep.* **5**, 10092; doi: 10.1038/srep10092 (2015).

## Supplementary Material

Supporting Information

Supporting Information

Supporting Information

Supporting Information

Supporting Information

Supporting Information
