# Supplementary material for: Comprehensive analysis of the T-cell receptor beta chain gene in rhesus monkey by high throughput sequencing
Source: Sci Rep. 2015 May 11;5:10092. doi: 10.1038/srep10092 (PMC4426732; doi:10.1038/srep10092)
Supplement: Supporting Information [file srep10092-s1.docx]

Comprehensive analysis of the T-cell receptor beta chain gene in rhesus monkey by high throughput sequencing

Running Title: Rhesus monkey TCRbeta immune repertoire sequencing

^1^Zhoufang Li^#^, ^2^Guangjie Liu^#^, ^1^Ying Tong^#^, ^1^Meng Zhang, ^3^Ying Xu, ^2^Li Qin, ^3^Zhanhui Wang, ^2^Xiaoping Chen*, ^1^Jiankui He*

^1^Department of Biology, South University of Science and Technology of China, Shenzhen 518055, China

^2^State Key Laboratory of Respiratory Disease, Center for Infection and Immunity, Guangzhou Institutes of Biomedicine and Health, Chinese Academy of Sciences, Guangzhou 510530, China

^3^Department of Infectious Diseases and Hepatology Unit, Nanfang Hospital, Southern Medical University, Guangzhou 510515, China

**Author Contributions**

#Authors contributed equally to this work

Design the project, JKH, XPC, ZFL; Perform the experiments: ZFL, GJL, MZ, YX; Data Analysis: YT, ZFL, LQ. Write and the manuscript: ZFL, YT, JKH, ZHW.

*Corresponding authors. E-mail: [he.jk@sustc.edu.cn](mailto:he.jk@sustc.edu.cn) (JKH); [chen_xiaoping@gibh.ac.cn](mailto:chen_xiaoping@gibh.ac.cn) (XPC)

**Supplementary Figures**

**
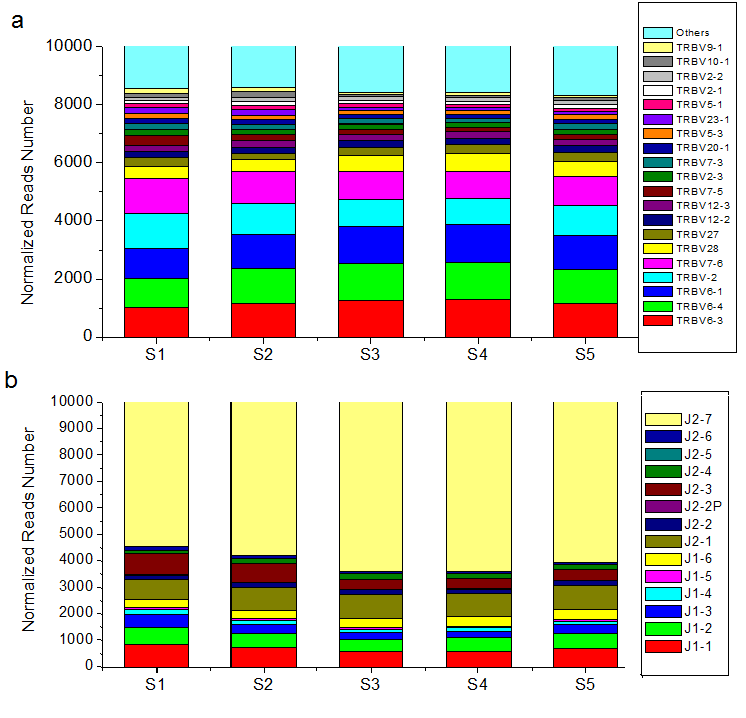
**

**Figure S1. V gene and J gene usage in the second monkey.** The genomic DNA from the second monkey was split into 5 separated tubes (S1 to S5) for multiplex PCR amplification and sequencing library construction. After shallow sequencing of these 5 samples, the V gene usage **(a)** and J gene usage **(b)** in 5 samples are analyzed. The pattern of V and J gene usage from different replicates are very similar.

**
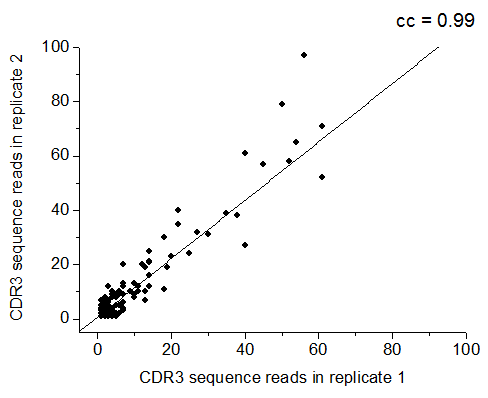
**

**Figure S2. CDR3 sequences correlation in two replications.** Each data point represents the abundance of a CDR3 sequence in two replications (sample S3 and S5). The correlation coefficient equals to 0.99, indicating good reproducibility.

**
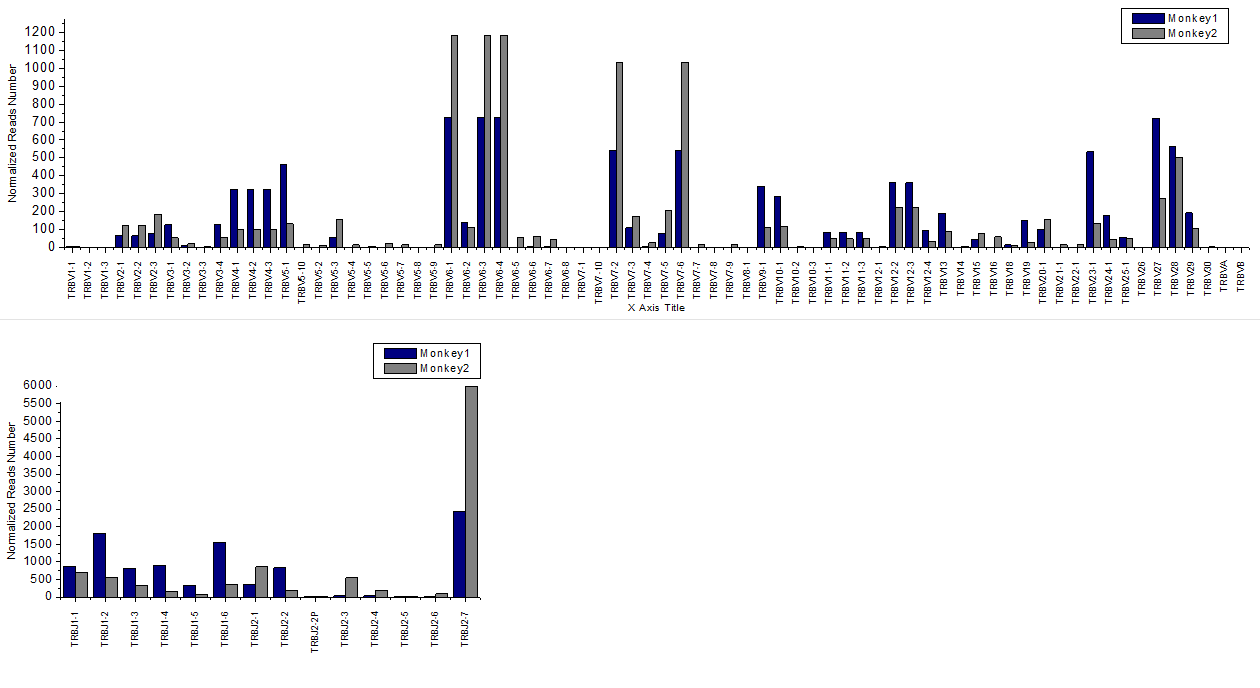
**

**Figure S3. V gene and J gene usages in two different monkeys.** A second monkey was employed in the experiment for primer set validation. Normalized reads in two monkeys are compared. Similar V and J gene are used in two different monkeys, however, the copy number of specific V gene or J gene usage in T cells are different, which indicates the variation of immune repertoire in different individuals.


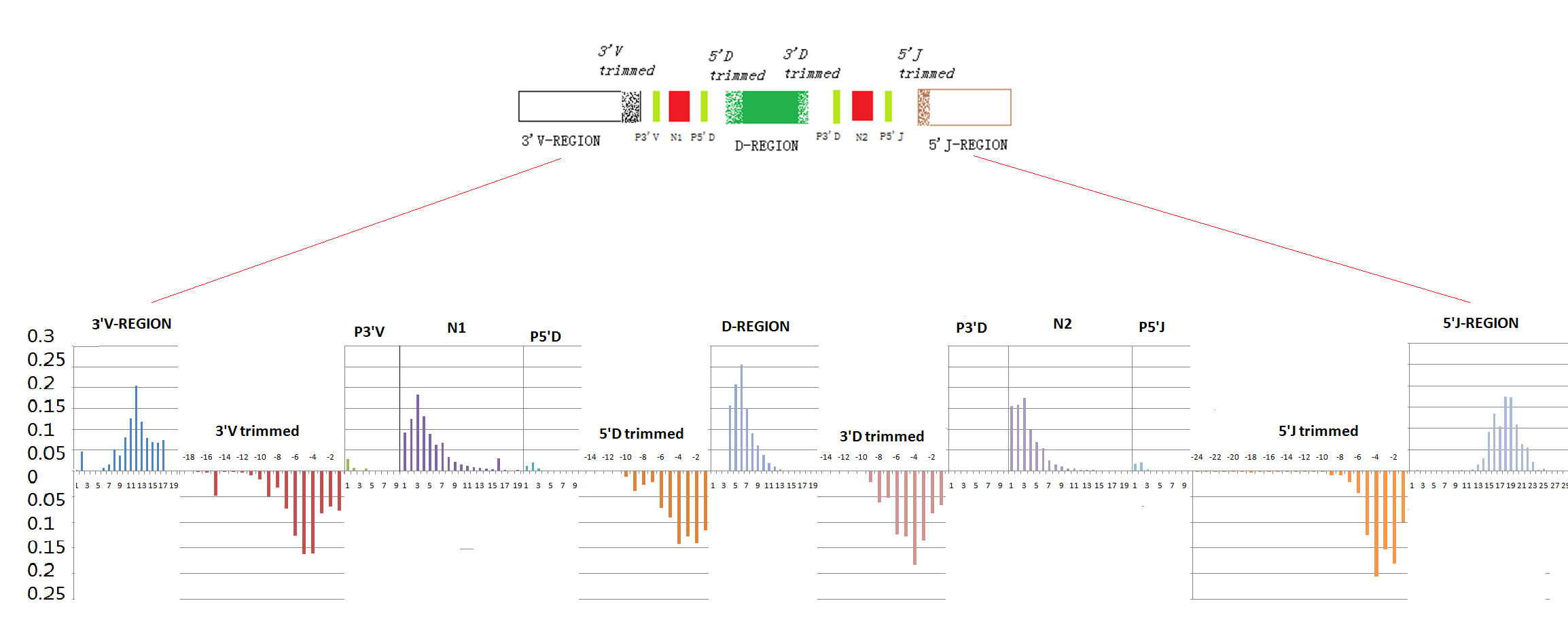


**Figure S4 2D plot of junction analysis of CDR3.** (a) Schematic diagram of 13 sections of the junction region. (b) The length distribution of insertions and deletions in 13 sections of the junction region. The x-axis is the 13 sections of the junction region and the y-axis is the frequency of the length distribution added or deleted in each section during the recombination process.


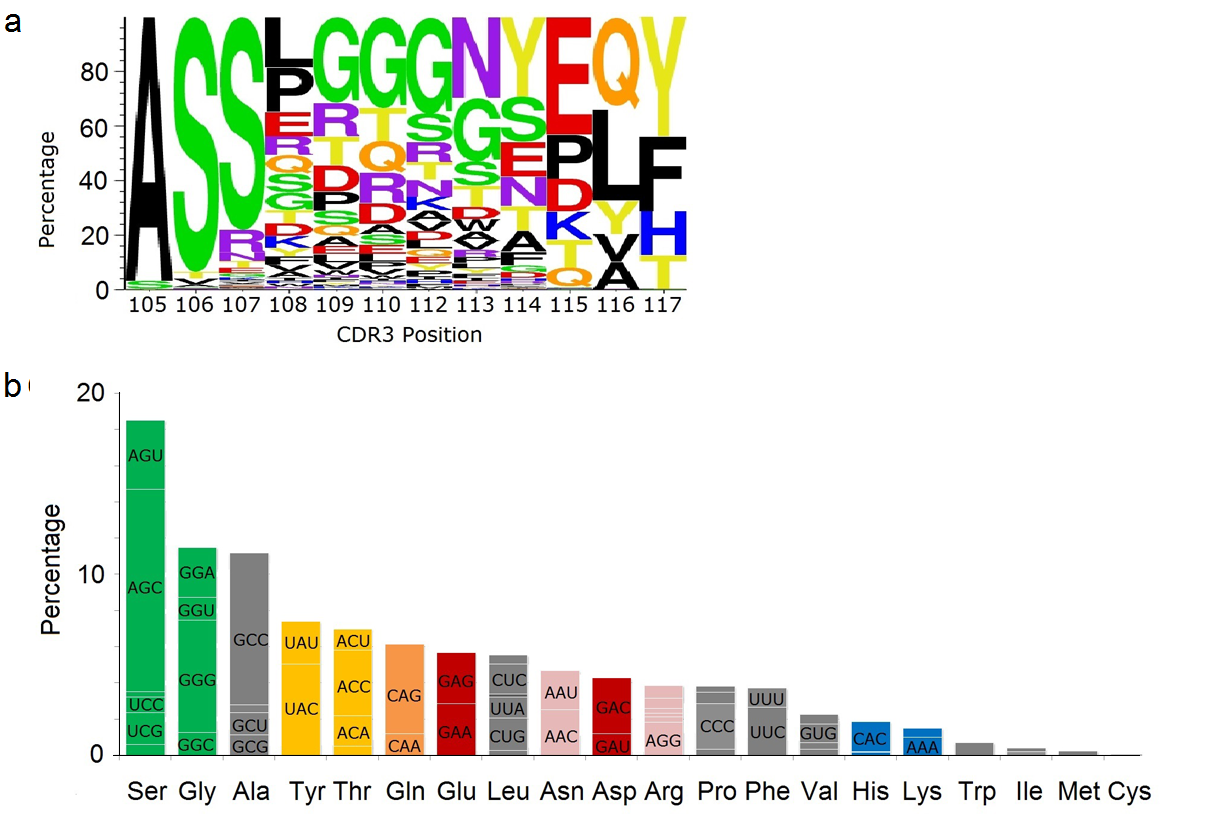


**Figure S5** (a) The most frequently observed length is 12 amino acids. For the subset of clonotypes with 12 amino acid CDR3 sequences, we created logos for the amino acid composition. (b) The frequency of codon usage for each amino acid in the CDR3


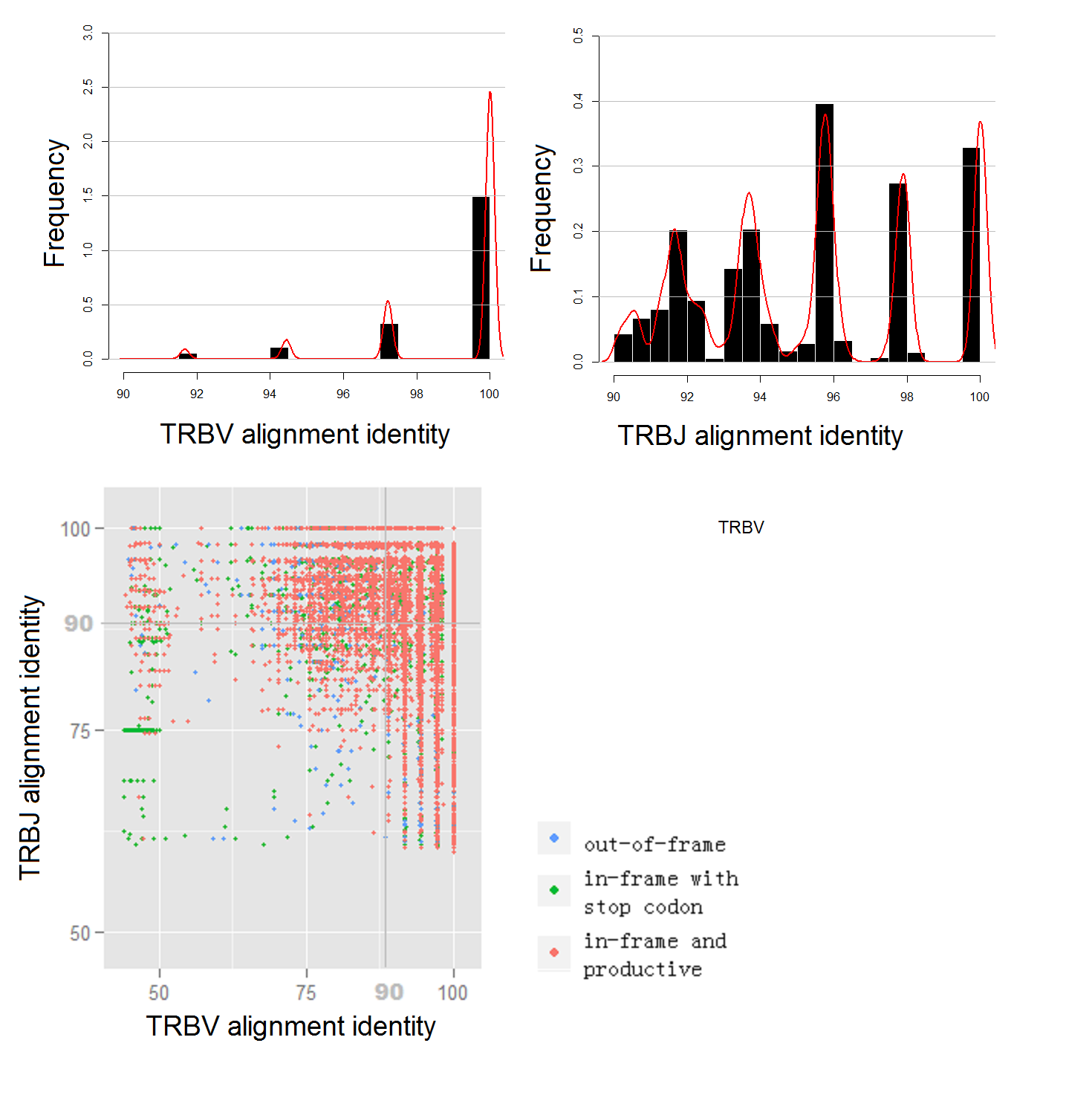


**Figure S6 2D density distribution of V/J gene alignment identity** When align our total 1,694,933 reads to references TRBV, TRBJ and TRBD genes, 1,264,773 (74.6%) reads can be arranged to their own VDJ combination with identity>60%, and 785,397 (46.3% of total) reads can be aligned to reference with identity>90%. There are 64 V references, 14 J references (including one pseudogene) and 2 D references, while we identified 57 V references, 13 J references and 2 D references with identity>90%.


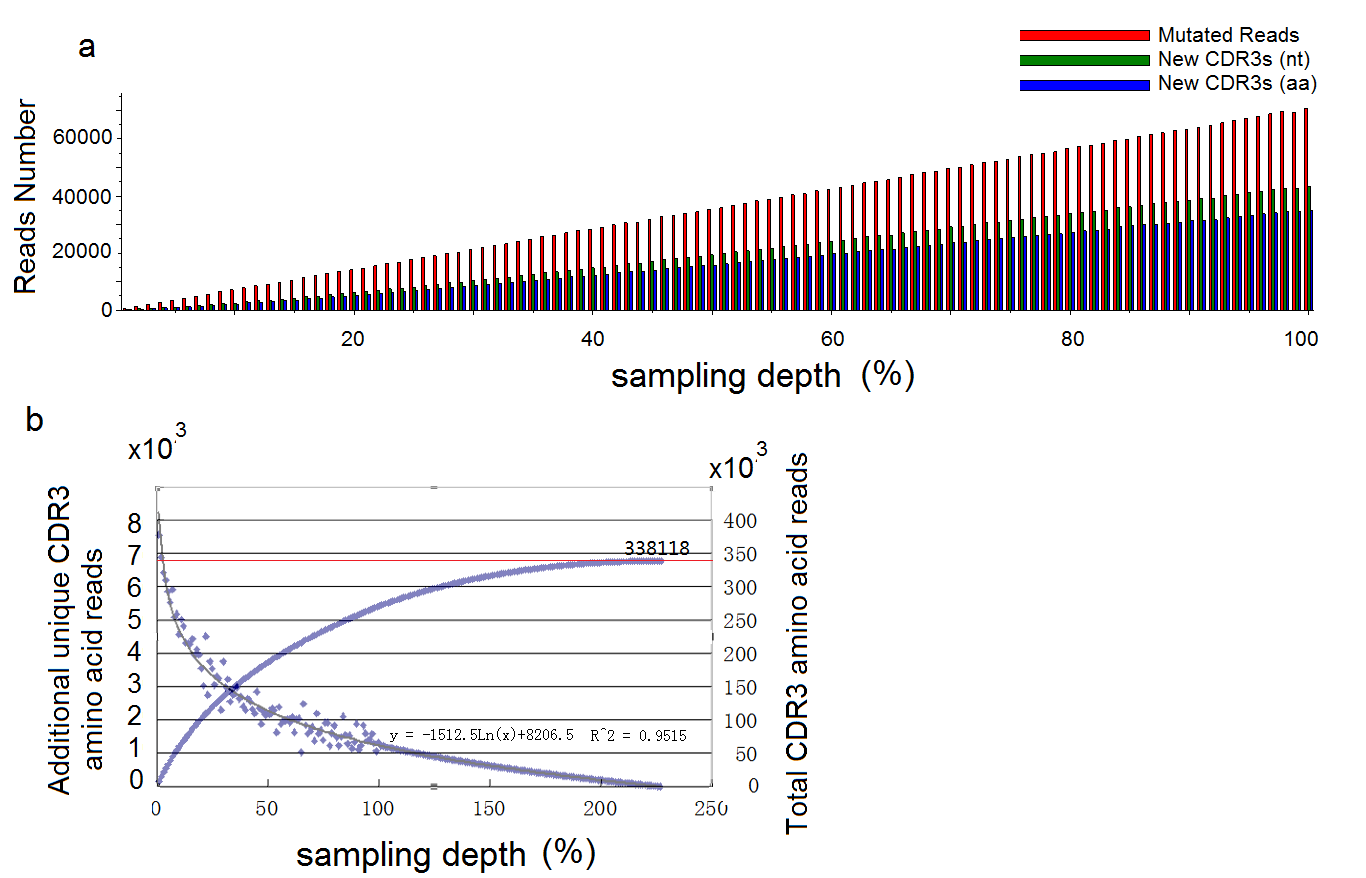


**Figure S7 Evaluating the effect sequencing error on the size of immune repertoire.** Plasmid sequences were used to estimate that 4.9% of CDR3 sequences contained one or more errors. The errors will artificially increase the size of immune repertoire, resulting in overestimation of diversity. To evaluate the influence of sequencing error on diversity, we artificially added new errors to the raw data in a computer simulation, run the sampling-resampling technique. By doing so, we can estimate to what extent the added sequencing errors will increase the diversity of immune repertoire. Each nucleotide was treated separately, having a chance (0.186%) to mutate into different base, corresponding to 4.9% errors per read. (a) A total of 70,549 reads were artificially mutated, resulting in 34,909 new CDR3 amino acid sequences. (b) After sampling-resampling, we estimated the size of TCR CDR3 repertoire by the new dataset. We got 338,118 CDR3 amino acid sequences. Therefore, in our simulation, by adding 4.9% errors to the original data, the diversity of TCR increased 1.29 times.

**Table S1 Forward and Reverse primers for TCRB of Rhesus Monkey**

| **V gene forward primers** | |
| --- | --- |
| TRBV1-1*01 | GCGCTGCAGCCAGAAGACTC |
| TRBV10-1*01 | TCTGCTGCCTCCTCCCAGAC |
| TRBV11-1*01 | CCTGCAGAGCTTGGGGACTC |
| TRBV12-2*01 | CCCTCAGAACCCAGGGACTC |
| TRBV2-1*01 | TCCACAAAGCTGGAGGACTC |
| TRBV3-1*01 | TCCCTGGAGCTTGGTGACTC |
| TRBV4-1*01 | GCCCTGCAGCCAGAAGACTC |
| TRBV5-1*01 | ACCTTGGAGCTGGGGGACTC |
| TRBV6-1*01 | TCGGCTGCTCCCTCCCAGAC |
| TRBV7-2*01 | CGCACAGAGCAGGGGGACTC |
| TRBV9*01 | TCTCTGGAGCTGGGGGACTC |
| TRBV13*01 | TCCTTGGAGCTGGGGGACTC |
| TRBV14*01 | AGTCCGGTATGCCCAACAAGC |
| TRBV15*01 | TGCTTTCTTGACATCCGCTCACC |
| TRBV16*01 | GCTACGAAGCTGAAGGATTC |
| TRBV18*01 | CAGGCAGAGCAAGAAGACTC |
| TRBV19*01 | TCAGCCCAAAGGAACCCAAC |
| TRBV20-1*01 | AATGCCCATCCTGAAGACAG |
| TRBV21-1*01 | AAGAGATTTTCAGCCCAATGTCCC |
| TRBV22-1*01 | TGAAGGCTACAGTGTCTCCCG |
| TRBV23-1*01 | TCCTCGGAACCAGGAGACAC |
| TRBV24-1*01 | TCTGCCACCCCCAACCAGAC |
| TRBV25-1*01 | TCTGCCAGCCCCTCACACAC |
| TRBV27*01 | TCGCCCAGCCCCAGCCAGAC |
| TRBV28*01 | TCCGCCAGCACCAACCAGAC |
| TRBV29-1*01 | AACACGAGCCCTGAAGACAG |
|  |  |
| **J gene reverse primers** |  |
| >TCRBJ2-6*01 | CCGAAAGTCAGGACGCTGGC |
| >TCRBJ2-3*01 | CTGGGCCAAAATACTGCGGATC |
| >TCRBJ2-5*01 | GGAGCACGCAGAGGTGGAAGC |
| >TCRBJ2-4*01 | CGCCGAAGTACTGAGTGTTTTGG |
| >TCRBJ1-6*01-02 | CCGTCACAGTGAGCCTGGTCC |
| >TCRBJ2-7*01 | TATGACTGTGAGCCTGGTGCCC |
| >TCRBJ1-2*01 | CTACAACAGTTAACTTGGTCCCTGAACC |
| >TCRBJ1-4*01 | CCAAGACAGAGAGCTGGGTTCCA |
| >TCRBJ1-3*01 | CTACAACAGTGAGCCGACTTCCCTC |
| >TCRBJ1-1*01 | CTAAAACTGTGAGTCTGGTGCCTTGTC |
| >TCRBJ2-2*01 | GCACGGTCAGCCTAGAGCCTTC |
| >TCRBJ2-1*01-02 | GAGCCGTGTSCCTGGCCCAA (S=C/G) |
| >TCRBJ1-5*01 | GGAGAGTCGAGTGCCATCTCCA |

**Table S2 Correlation between V/J gene usages of different samples**

| Correlation between V gene usages of samples | | | | |
| --- | --- | --- | --- | --- |
|  | S1 | S2 | S3 | S4 |
| S2 | 0.985338605 |  |  |  |
| S3 | 0.960397409 | 0.987215205 |  |  |
| S4 | 0.953045845 | 0.982510915 | 0.998375304 |  |
| S5 | 0.974618119 | 0.992217629 | 0.99704845 | 0.994417798 |
|  |  |  |  |  |
| Correlation between J gene usages of samples | | | | |
|  | S1 | S2 | S3 | S4 |
| S1 |  |  |  |  |
| S2 | 0.998435738 |  |  |  |
| S3 | 0.992563919 | 0.996966943 |  |  |
| S4 | 0.992936126 | 0.997046781 | 0.999877551 |  |
| S5 | 0.99466048 | 0.997773653 | 0.999513048 | 0.999509587 |

**Table S3 Reads number of 64 TRBV gene segments**

**Table S4 Reads number of 13 TRBJ gene segments**

**Table S5 Reads number of 2 TRBD gene segments**

**Table S6 Reads number of V-D-J recombinations**

**Table S7 Summary of data**
